# Exploring Therapists’ Approaches to Treating Eating Disorders to Inform User-Centric App Design: Web-Based Interview Study

**DOI:** 10.2196/68846

**Published:** 2025-05-06

**Authors:** Pamela Carien Thomas, Pippa Bark, Sarah Rowe

**Affiliations:** 1 Epidemiology and Applied Clinical Research Department University College London London United Kingdom; 2 UCL Cancer Insititute London United Kingdom

**Keywords:** eating disorders, binge eating, bulimia, anorexia, qualitative research, mental health, smartphone apps, digital interventions, eHealth, mobile health, artificial intelligence

## Abstract

**Background:**

The potential for digital interventions in self-management and treatment of mild to moderate eating disorders (EDs) has already been established. However, apps are infrequently recommended by ED therapists to their clients. Those that are recommended often have poor engagement and user satisfaction, leading to unsatisfactory outcomes. Barriers to recommendation include patient safety, data privacy, and a perception that they may not be effective. Many existing interventions have limited functionality or do not differ much from manual cognitive behavioral therapy (CBT) or self-help books, which may not adequately support the therapeutic process or sustain user engagement.

**Objective:**

This study aims to explore the perspectives of therapists who support people with mild to moderate EDs in the community, exploring their existing treatment approach and how an ED app might fit in the treatment pathway alongside treatment.

**Methods:**

Semistructured web-based interviews were completed with ED therapists in the United Kingdom. Participants were recruited from First Steps ED, a specialist community-based ED service, and Thrive Mental Wellbeing, a workplace mental health provider. Five main themes were covered: (1) therapists’ treatment approach, (2) how therapy was implemented in practice, (3) strategies for engaging and motivating clients, (4) perspectives on a potential ED app, and (5) suggestions for app content and design. A structured thematic analysis was validated by 2 researchers.

**Results:**

Overall, 12 ED and mental health therapists (mean age 28.7, SD 7.3 y; female therapists: n=7, 58%; male therapists: n=5, 42%) participated. Therapists dealing with complex ED issues went beyond traditional CBT using additional therapeutic techniques and a flexible, person-centered approach to treatment. This included engagement and motivational strategies to support the client, elements of which could be mirrored in an app. Therapists identified the therapeutic relationship as key to success, which might have been hard to replicate in an app. They saw the potential for evidence-based apps across all stages of the treatment pathway. The need to address safeguarding, data privacy, and the potential for triggering content within the app was vital.

**Conclusions:**

This study advanced our understanding of how to design and develop clinically safe, evidence-based ED apps that can complement therapy by extending the continuity of care and the self-management and psychoeducation of clients. It emphasized integrative, adaptive CBT that incorporated other therapeutic approaches based on individuals’ needs, which could be replicated in an app, as could the strategies to support engagement and motivation. It gave a cautious yet optimistic perspective on the potential integration of apps into ED treatment across all stages of the treatment pathway, from pretreatment maintenance to posttreatment maintenance. It highlighted various concerns that could be addressed and potential limitations, such as the therapeutic relationship, while recognizing the growing potential of apps with rapid technology and artificial intelligence advancements.

## Introduction

### Background

Eating disorders (EDs) are complex mental health conditions characterized by severe disturbances in eating behaviors and related distressing thoughts and emotions. They can have a significant impact on individuals’ psychological, physical, and emotional well-being and are related to serious health consequences, such as mortality, infertility, osteoporosis, as well as a reduced quality of life [1,2]. Most people with mild to moderate EDs are not getting help due to the stigma and shame associated with their condition [1] and a high demand for health services, which has escalated since the COVID-19 pandemic [3]. Those with milder forms of EDs are less likely to receive support as they are considered at a lower risk, hence lower priority, despite the importance of early intervention [4]. Those who are deemed ineligible for public health care or who are on a waiting list for treatment can seek support from a private health care service provider or an ED patient group, such as Beat or First Steps ED in the United Kingdom. However, barriers such as cost, lack of awareness, or reluctance to talk to a person remain [1], and these services have limited capacity.

Apps are a promising tool to support the management of people with mild to moderate EDs by overcoming some of these barriers to treatment [5]. Apps need to be trustworthy, safe, and effective [6,7]; hence, evidence-based design and clinical involvement in their development are crucial. Fairburn and Rothwell [8] stressed the importance of professional involvement in translating evidence-based treatment, in particular cognitive behavioral therapy (CBT), into digital interventions to ensure therapeutic integrity of the digital format [9], although this has rarely been the case with ED apps.

Systematic reviews of randomized controlled studies have provided robust evidence for the effectiveness of digital interventions in reducing ED symptoms in the short term, with some studies demonstrating effectiveness at long-term follow-up. However, evidence for apps is still limited [10], with high dropout rates [11,12] and significant engagement challenges (eg, uptake, ongoing use, and attrition) making the suitability of apps in real-world settings particularly relevant. Evidence from qualitative studies suggests that, while these interventions have received some positive feedback from potential users [13,14] there is significant room for improvement to make them more appealing and engaging. Clinicians have also expressed concerns and barriers to recommending apps, including a lack of personalization, patient safety, and data security and privacy [13]; this has resulted in limited uptake [5]. Many existing interventions differ little from written self-help programs [14] or simple digitized versions of manual CBT [15]. Intervention design should be based on what works in traditional therapy while capitalizing on the advantages offered by a digital format [7,14] to support engagement and effectiveness.

One recommended approach to improve engagement and effectiveness of apps is to conduct qualitative research with clinicians to inform intervention planning, design, and development [16-19]. Few qualitative studies have explored the in-depth views and experiences of therapists regarding apps, and they have been typically involved at a final evaluation stage of the development process [20,21]. One recent exploration on the perspective of German clinicians on digital ED interventions and their integration into routine care [13] focused on barriers and enablers to their use. It was positive about the potential to improve access to treatment, provide beneficial monitoring and engagement tools, and act as a useful adjunct to therapy. However, clinicians expressed concerns, including losing the benefits of the therapeutic relationship with their clients; how patient data might be used, stored, and accessed; and whether apps could be personalized enough to meet individual patient’s needs, especially in the more severe cases. The study did not explore how to design digital ED interventions that were acceptable to clinicians, how these barriers could be overcome, or how they could be made more engaging for users. It also did not focus on apps specifically.

Our study explored the views of therapists working in a community setting and how they delivered effective treatment to people with mild to moderate EDs. This study aimed to bridge the gap between traditional clinical treatment approaches and apps with a focus on how these methods can inform app design. CBT is the “gold standard” for eating treatment (National Centre for Clinical Excellence) and frequently forms the basis for app design, with some apps hardly differing from digitized manual CBT. This study challenges the assumption that digitized traditional CBT is appropriate for app development by exploring how therapists deviate from standard CBT and understanding the justifications for this to inform the development of an effective and engaging app.

### Objectives

The primary objectives of this study were to understand the following: (1) how therapists currently supported people with mild to moderate EDs in the community; (2) how therapists motivated and engaged their clients in treatment; (3) where an app might fit in the treatment pathway, who it is most suited for, and what concerns needed addressing; and (4) how therapists envisaged app content and design.

## Methods

### Overview

In total, 12 web-based semistructured interviews, ranging from 45 to 60 minutes, were completed with ED therapists in the United Kingdom. Participants were asked about how they supported their clients with ED and their perspectives on a proposed ED app for people with mild to moderate EDs (Multimedia Appendix 1 provides the interview guide). This qualitative study was nested within a broader research program on the development and evaluation of an ED app module for people with mild to moderate EDs.

The COREQ (Consolidated Criteria for Reporting Qualitative Research) checklist was followed [22]. This checklist helped to clarify the procedures, analysis, and interpretation.

### Participants

Participants were therapists, counselors, and a psychiatrist working with a patient group with an ED (First Steps ED) and a mental health service provider (Thrive Mental Wellbeing) across the United Kingdom. This enabled a comprehensive understanding of treatment approaches for people with mild to moderate EDs and identified potential benefits, risks, and concerns relating to apps. They had to have been in their role for at least 3 months and completed their compulsory training. The term “therapist” is used throughout this paper as a blanket term to cover the range of professional experiences.

### Recruitment

We targeted therapists who worked in a community setting, as their patient population may be more reflective of those who would be most suitable for an app and for whom self-management support may be an appropriate option. Convenience sampling was used with participants selected based on their availability and willingness to participate, given therapists’ time constraints. Random sampling was not required, given the aim of this study was to develop meaningful insights and further our understanding across a range of professionals, without aiming for generalizability. Given the exploratory nature of this study, the sample was not required to be representative, so a relatively small sample size was deemed acceptable [23].

Recruitment took place via First Steps ED, a specialist ED support service, and Thrive Mental Wellbeing, an industry collaborator providing mental health support via employers. To maintain rigor and relevance, team managers at both organizations facilitated the recruitment process, ensuring that only therapists with relevant ED experience or training were invited to participate. At the time of recruitment, Thrive Mental Wellbeing had approximately 25 therapists, though not all specialized in ED treatment, and First Steps ED had 18 trained ED therapists and counselors. By engaging 2 distinct yet complementary organizations, we ensured a breadth of perspectives by capturing insights from therapists working within a dedicated ED service (First Steps ED) and those delivering broader workplace-based mental health support (Thrive Mental Wellbeing).

Interviews were online and audio and video recorded (via Microsoft Teams) between January and February 2024. Field notes were taken after the session to capture important points and initial reflections.

### Materials

Semistructured interview questions were used to guide the interview discussion. The interview guide was developed by the primary researcher (PCT) in consultation with a public and patient involvement group from First Steps ED comprising the research manager, clinical service manager, and 2 specialist support officers. Questions focused on the therapists’ treatment approach, how they applied this in practice, the target group for the app, engagement and motivation, and the design and functionality of the app (Multimedia Appendix 1 provides the interview guide).

### Data Analysis

The audio recordings were transcribed using Microsoft Teams and then anonymized and checked for accuracy by the primary researcher (PCT). The scripts were then imported into NVivo (version 12; Lumivero) [24] for analysis. The analysis followed a structured thematic approach based on the approach by Clarke and Braun [25]. The primary researcher created initial codes across all transcripts and then reviewed and consolidated these into an initial coding framework of main codes and subcodes in an iterative process. The second coder (PB) familiarized herself with 25% of the data and independently generated initial codes and subcodes iteratively. The 2 coders refined their results in several iterations until agreeing upon a coding framework against which all the interviews were coded. The primary researcher consulted with a public and patient involvement team at First Steps ED to validate the final coding framework. A codebook was generated to support the implementation of the coding framework across both coders and ensure reliability. Given the complexity of the topic and interdependency of some of the themes, it was possible for the same quote from participants to be used against more than one theme and subtheme. We did not aim for saturation, as the approach by Clarke and Braun [25] prioritized generating meaningful insights over complete data coverage. Because data were relatively homogeneous across interviews, common themes and subthemes were easily identifiable.

### Reflexivity Statement

The primary researcher was a female researcher with experience and training in qualitative research methods. She reflected on any potential biases during the coding, such as an assumption that apps have the potential to address existing service gaps and help support people with EDs who may currently not be getting help. She remained receptive to the insights and experiences shared by ED therapists, including participants who were more skeptical about the role of apps in treatment, and made sure to probe all interviewees about their potential concerns regarding apps.

### Ethical Considerations

This study received ethics approval from the University College London Research Ethics Committee (23943/001). The research adhered to ethical guidelines for qualitative studies involving human participants.

#### Informed Consent

Participants received an information sheet outlining the aims and objectives of the study. This informed them of the study’s purpose, voluntary nature, and the right to withdraw at any time without consequence. They provided consent via a digital consent form in a secure electronic data platform, REDCap (Research Electronic Data Capture; Vanderbilt University) before the participation and reconfirmed their consent at the start of the session. After providing informed consent, therapists participated in one-on-one web-based interviews from their workplace. No participants dropped out after giving their consent. Participants did not know the researcher, although there was friendly email exchange before each session to establish a rapport, clarify session objectives, and address any participant queries. Participants were aware of the researcher’s role and motivation to explore how an ED app could be developed to support treatment, aligned with the study’s objectives.

#### Privacy and Confidentiality

Baseline characteristics of participants were collected via Microsoft Forms, hosted on a secure University College London server. All study data were securely stored, and standard anonymization protocols were followed after transcription. Identifying information was removed to maintain participant confidentiality. Only the research team had access to the data, and no personally identifiable information was included in this paper or the multimedia appendices.

#### Compensation

Participants were not offered financial or material compensation for their involvement in the study. Their participation was motivated by a shared commitment to improving access to treatment for individuals with EDs.

#### Participant Identifiability

No personally identifying details of participants are included in this paper or the multimedia appendices. In cases where quotations from transcripts are presented, they have been deidentified to prevent recognition of individual participants.

## Results

In total, 12 interviews were completed and lasted an average of 50 (SD 6; range 35-60) minutes.

### Participant Characteristics

In total, 8 therapists, 3 counselors, and 1 psychiatrist took part. Participants were aged between 21 and 52 (mean 28.7, SD 7.3) years. Most (11/12, 92%) therapists were trained in CBT and had a range of experience and training, including dialectical behavior therapy and acceptance and commitment therapy (ACT). All were educated till the master’s level (10/12, 83%) or had higher education (2/12, 17%).

Moreover, 50% (6/12) of the participants had direct experience of working alongside apps, allowing them to speak from experience in terms of how an app could work alongside treatment, and 50% (6/12) did not. Most participants were positive (10/12, 83%) that apps had the potential to be a valuable tool in the treatment process and felt that they might or were likely to recommend a person-centered app to their clients (8/12, 67%). However, 25% (3/12) of the participants agreed with the statement that they had significant concerns about their use in treatment (refer to Multimedia Appendix 2 for the baseline characteristics).

Five themes were covered. The first 3 themes provided crucial context for informing app development by providing an in-depth exploration of how treatment was delivered to help identify elements that could be prioritized and adapted. These included therapists’ treatment approach, how therapy was implemented in practice, and strategies for engaging and motivating clients. The subsequent 2 themes focused explicitly on the app itself and explored perspectives on a potential ED app and suggestions for app content and design. Therapists discussed where an ED app might fit within the treatment pathway, who it would be suitable for, and any potential concerns.

### Theme 1: Therapists’ Treatment Approach

#### Overview

The theoretical treatment approach used by all was grounded in CBT, an evidence-based path to recovery; however, in dealing with the complexity of EDs, it went beyond the traditional form. Therapists emphasized personalization in treatment, combining additional therapeutic techniques, such as ACT, compassion-focused therapy, and psychodynamic approaches.

#### CBT as an Evidence-Based Approach

CBT was described as the “gold standard” treatment covering a structured, goal-oriented approach that helped individuals identify and challenge negative thought patterns and behaviors, making it especially effective for addressing disordered eating behavior. Therapists appreciated the practical, skill-oriented approach of CBT and the provision of a toolkit of resources and concrete tools to change ED behaviors. A strength of CBT was the ability to address key features of EDs, such as cognitive distortions around food and body image and avoidance of feared foods:

A lot of people with eating disorders struggle with like perfectionism and black and white thinking. It’s that very rational and everything has to be kind of logical sort of thinking in some ways and and obviously CBT helps with that, it helps change those ways of thinking.

Therapist 8

Firstly, giving them tools that will help reduce anxiety around food cause obviously that’s really important. And then very gradually introducing and perhaps foods that they might deem or a food they might deem unsafe, and they avoid.

Therapist 11

It was suggested that an app might be useful in providing access to CBT-type resources for those who were unable to access therapy:

There’s so many people who don’t get access to therapy and don’t get access to support, to have something that’s in your pocket, that’s just really convenient would be really useful...there’s a couple of websites where you can find typical CBT style kind of interventions and worksheets and if you could just have those accessible to people.

Therapist 8

However, therapists noted CBT limitations for this population. The structured format, including homework and behavioral tasks, did not suit everyone, particularly those with neurodivergent conditions. For example, individuals with attention-deficit/hyperactivity disorder (ADHD) might require shorter, more creative sessions to stay engaged, and the rigidity of traditional CBT could be overwhelming. Therapists also observed that CBT assumed individuals were willing and able to engage with cognitive restructuring and behavioral change, which might not be true for those early in their recovery or resistant to structured approaches. Finally, CBT was not seen as appropriate for deeper emotional issues, such as trauma:

It’s not ethically OK to...start trauma work in session 8 and leave them after two sessions.

Therapist 7

I think there is a limit to CBT personally, because essentially there’s always deeper issues that lead into the behaviors and the thoughts and the emotions.

Therapist 5

#### Person-Centered Care

While CBT principles were followed, they were adapted to fit the client’s situation to a person-centered approach aligning with the client’s preferences and readiness:

It’s good to have a protocol as a guide to kind of give you some structure, but you can obviously amend it...this is where it comes into being more person-centred.

Therapist 10

Adaptability was key to ensuring therapy remained relevant to everyday ED challenges and individual differences:

We can adapt things to make it fit the service user and we follow that lead a little bit to be honest as well.

Therapist 4

There was recognition of the potential role of apps to provide different content adapted to individual clients:

Provide certain content based on certain personal characteristics...to have different sort of journeys through the things that would be the most likely to be of interest to you.

Therapist 2

#### Complementary Treatment Approaches

Complementary treatment approaches addressed emotional and psychological needs beyond the scope of CBT:

The integration of these complementary approaches added emotional depth to the treatment process...[they] delve into the deeper issues...that led to the eating disorder.

Therapist 8

The approaches most commonly mentioned were ACT [26] and compassion-focused therapy. ACT was seen as helping individuals accept their thoughts and emotions, encouraging them to commit to actions aligned with their values and not struggle against them. It was seen as helping clients link recovery to deeper, personal motivations and used to reduce self-criticism and cultivate self-compassion:

I integrate things like compassion-focused therapy...creating visualizations or safe spaces around compassion for self.

Therapist 7

Learning self-compassion was seen as particularly relevant to ED treatment, where clients often experienced shame and self-judgment, helping them develop a compassionate inner voice and offer a gentler, more empathetic approach to their issues:

It helps change the stories we tell ourselves.

Therapist 8

### Theme 2: How Therapy Was Implemented in Practice

#### Overview

The interviews underscored the importance of structured sessions that incorporated both standard therapeutic activities and flexible content addressing the unique aspects of each client’s situation and gave some insights into what could be mirrored in an adjunct app. Collaboration with other professionals was crucial, especially when symptoms were severe or complex.

#### Structure and Content of ED Therapy

Therapy typically included a set number of sessions, usually 10, especially when the National Health Service commissioned the patients. Key components included initial assessments, psychoeducation on nutrition and body image, and interventions, such as cognitive restructuring, behavior experiments, and relapse prevention.

The initial assessment developed trust and was designed to understand the client’s history and identify treatment goals. Sessions started with broad, open-ended discussions before narrowing to specific issues, allowing a thorough exploration of the client’s experiences. Some flexibility ensured treatment was kept relevant to the client’s immediate needs, whether addressing restrictive behaviors or navigating challenging social situations (refer to Multimedia Appendix 3 for the treatment stages).

Tools included food and thought diaries to guide the direction of therapy:

Sessions begin with reviewing that food and thought diary from the week and that can guide where the support will go for that session.

Therapist 11

Psychoeducation was a crucial part of therapy, where clients learned about nutrition and the physical effects of ED behaviors:

We go through things like the real food pyramid...talking about the effects of purging on the body.

Therapist 11

The structure included educating clients about societal influences on body image:

I delve into the impact of social media and diet culture on body image...breaking down whether what’s posted is a true snapshot of people’s lives.

Therapist 11

#### Tailoring Sessions to Individual Needs

As clients came with different histories, experiences, and cognitive styles, therapists emphasized the importance of adapting to personal preferences, specific symptoms, and past experiences. For example, therapists sought to understand what had or had not worked in the past to avoid repetition and to build on strategies that resonated with the client:

So with a lot of adults, you’ll often find that they’ve had support in the past and they will sit there and tell you that I have tried this before and that didn’t work for me. I’ve tried this and I really enjoyed that, and I got a lot from that.

Therapist 12

Male participants faced specific challenges and stigma related to their ED, which needed to be addressed in treatment:

A lot is you will lose your periods and it’s like men won’t...making sure we’re talking about testosterone levels...I’m working with a young gentleman at the minute that is infertile.

Therapist 4

The adaptability of the therapeutic process was evident in session planning:

We wouldn’t sit there with someone and say...we need to do this in Session 5...we are able to have that flexibility and look at what they are struggling with and really just try and help that person how they need help that week essentially.

Therapist 12

Another key aspect was the continuous evaluation of the client’s state, which allowed therapists to adjust the content and timing of the therapeutic plan:

If we can actively see, OK, you’ve you’ve binged quite a bit this week. Let’s try and focus on that a little bit in some of our future sessions.

Therapist 12

If a client was struggling with body image issues, the focus would shift to body acceptance. This involved reframing how they thought about their bodies, helping them move from viewing their bodies traditionally in aesthetic terms to appreciating their functionality:

What does that body part do for you? Does how it look change its function?

Therapist 11

Therapists recognized that clients worked at different speeds, allowing clients to explore at their own pace and determine the best course of action for themselves:

I never forced them to do anything...if they don’t feel ready, that’s fine.

Therapist 3

#### Collaboration or Referral to Other Specialists

Clients were referred to specialists when they presented with severe symptoms, such as very low BMI or comorbid mental health conditions, or had needs beyond the therapist’s expertise requiring more intensive medical or psychiatric support:

If someone is struggling with let’s say suicidal ideation for example, we make sure somebody’s got the general mental health support in place.

Therapist 8

There was a collaboration with additional professionals, and clients were provided with access to support networks and group support sessions. This included expert nutritional advice and support in meal planning:

Seeing a dietitian or nutritionist who was working with them, who’s working very specifically with him about creating some sort of food plan.

Therapist 5

For clients with more severe symptoms, therapists worked within a multidisciplinary team that assessed and planned the best course of action for each client:

If they’re a little bit more severe and it’s a little bit more complicated...we will then take them to an MDT meeting and where we just got off to discuss the issue in the case with the rest of the team and kind of just decide between us what’s the best course of action for them.

Therapist 8

For those who progressed with therapy, help was provided at the end of treatment to help clients transition to less intensive “step-down” support services, such as group sessions or befriending programs. The final sessions involved developing relapse prevention plans, where options for ongoing support were discussed:

We put in things like exploring triggers and their support networks...making it clear there’s still support, just not one-to-one.

Therapist 8

This could also include referral to an ED app, which several therapists suggested could provide support at this difficult stage:

Really see it as as step-down option after treatment, a way to check in with themselves...because once you’ve left recovery, you’re on your own.

Therapist 4

### Theme 3: Strategies for Engaging and Motivating Clients

Engagement created a safe, supportive space where clients felt understood and valued, and motivation focused on helping clients recognize the need for change and overcome any resistance to change.

#### Therapeutic Relationship

The therapeutic relationship, defined by trust, empathy, and mutual understanding, was seen as fundamental to the success of therapy. Therapists emphasized the importance of rapport, empathy, and creating a safe space where clients felt heard and supported. Although therapists were open to the potential role of an app in treatment, they were skeptical about whether it could provide personalized support, encouragement, and empathy essential for successful treatment outcomes:

The relationship is everything in therapy. So I don’t really know how that transcribes into an app, and so for that reason I am a little bit wary of apps in a lot of ways.

Therapist 7

One therapist highlighted the importance of making the first session comfortable (“The first session is very relaxed, very chilled”; therapist 11) while the majority expressed the importance of creating a (“safe space”; therapist 7). Trust was fundamental, allowing clients to open up about sensitive issues and (“do the hard things”; therapist 2):

You’re trying to hold a safe space for them, allowing them to talk and be open...but also trying to challenge their identity and aspects of themselves.

Therapist 8

Creating a therapeutic alliance, showing congruence, showing unconditional positive regard and care for the service user that I’m working with to create a safe space for us to work on.

Therapist 5

Therapy was seen as a partnership, where therapists and clients worked together to set goals and navigate challenges. Clients had to feel confident that they would not be “told off” (therapist 7) or “judged” (therapist 1).

#### Client Empowerment

Empowerment focused on helping clients take ownership of their recovery, such as supporting goal setting and progress tracking. This also included setting boundaries, ensuring that clients realized responsibility for change lay within themselves. Guidance extended beyond sessions with strategies, such as mindfulness and self-reflection exercises, promoting independence and self-management. One therapist mentioned using an artificial intelligence (AI) tool to encourage self-reflection outside of sessions.

Therapy helped clients increase self-awareness and take control:

What makes therapy successful is towards the end...the user is more self-aware, they can almost hear their therapist making their reflections and challenges.

Therapist 5

I don’t take an expert role...although I’m providing techniques, what I am saying is, you are the person who will be able to affect these.

Therapist 7

#### Client Engagement

Customization of resources was important to support client engagement, with worksheets often adapted to make them more acceptable to their clients. This was particularly important for clients with specific needs, such as neurodiverse individuals with ADHD or autism who struggled with focus or traditional therapy methods:

I will sit there and literally create a resource...adding pictures or using bright colours.

Therapist 7

These types of techniques could be mirrored within an app:

If they have to log in, you know allowing customization of like the home page for example that you go to just then it’s a bit more inviting for them.

Therapist 11

Providing gentle challenges, along with regular feedback on progress and positive outcomes, was seen as key to keeping participants engaged in therapy:

Getting them to reflect on where they are, where they’ve been and what kind of path they’ve been on is really important.

Therapist 5

Keep focusing on the wins, you know, getting to pick out a couple of positives, positive things that happened this week or positive things they learned over the last three weeks or something like that.

Therapist 11

Complex tasks were broken down into manageable chunks to help clients engage in activities. Setting small, regular goals was seen as valuable in giving clients a sense of accomplishment, supporting ongoing engagement:

The absolute best homework is set your alarm, get up in the morning and do one task and that’s your homework for the week.

Therapist 7

These techniques, including goal setting and monitoring, were seen as easily transferable to apps:

Something in your phone that, for example, has your agreed goals.

Therapist 2

#### Addressing Motivation

Therapists used various techniques to enhance clients’ motivation and address ambivalence or resistance. Motivational exercises such as future visualizations helped clients envision the outcomes of their behaviors and set goals for change, highlighting potential consequences of their actions and motivating change:

We picture ourselves and the tree has two halves...one is if I continue with these behaviors, where will I be in one year, three years, five years?

Therapist 12

Therapists helped clients address resistance by exploring conflicting feelings about change, providing a safe space to discuss fears, and “to help them challenge or reframe their reservations” (therapist 5):

If I was to keep this eating disorder, what does that mean for myself? And then also get them to look at...if I was to lose and if I was to recover, what would that look like?

Therapist 8

Setbacks and lapses were discussed as learning opportunities rather than failures to help maintain motivation. Therapists encouraged a growth mindset, offering support and understanding while focusing on incremental progress:

We’re always learning...slipping backwards and forwards. The power is in those lapses; that’s the learning.

Therapist 7

Therapists highlighted that if a client was resistant to change, there was only so much they could do and the client might not have been ready for therapy yet.

### Theme 4: Perspectives on a Potential ED App

#### Overview

Perspectives on a potential ED app alongside therapy were generally positive. However, some therapists were skeptical, citing concerns about triggering content, safeguarding, and data privacy. There was also a critical view of the lack of moderation and poor effectiveness of existing ED apps and concerns about how well an app could personalize therapy or support a therapeutic relationship.

#### Value of an App

Therapists recognized the potential value of an app for ED treatment as a complementary tool to traditional therapy. It offered greater accessibility to psychoeducational content about EDs and a nonjudgmental space for users who were reluctant to engage in face-to-face treatment. It could also offer users a pretreatment, private space to explore their feelings and behaviors, potentially reducing their stigma and shame, especially for those initially hesitant to seek support:

You can educate yourself...without judgment because I know that a lot of people don’t come to therapy because they are afraid of judgement.

Therapist 1

Therapists commented on the advantage for clients to have educational materials and self-help tools to enhance their sense of independence and maintain continuity between therapy sessions:

An app could provide continuous support, be informative and assist with self-education.

Therapist 5

An app could also serve as a “follow-up” (therapist 11) to in-person therapy, helping clients track their progress and reinforce the concepts discussed during sessions, as well as a helpful “step-down approach” (therapist 9) at the end of therapy.

#### Who Would Be Most Suitable for an App?

Therapists felt an app would be best for individuals with mild to moderate symptoms, as they were at a lower risk and more likely to engage in treatment. It could work as a form of early intervention to prevent symptoms from worsening or for those who were no longer in a severe phase*.* Two therapists mentioned the potential for a recovery tool for patients with more severe symptoms to help prevent relapse:

After people have been in the more moderate to severe category, they’ve had support and then they’re sort of stepping away from that support, I think it will be a really useful tool to help people continue on with what they’ve learned.

Therapist 9

#### Where Would an App Fit in Treatment Pathway?

Regardless of severity, therapists highlighted that users would need motivation to use an app. They emphasized that insights, personal motivation, and readiness to change were key factors in determining its suitability:

It has to be a user who’s committed to the change or committing to wanting to change.

Therapist 5

Therapists saw an app as beneficial at various points in the treatment journey, from before treatment, when individuals sought to understand their behaviors but were uncomfortable discussing them, to being a valuable resource in recovery. There was consensus that an app could complement therapy.

Several participants suggested that an app could be especially valuable for individuals on waiting lists for therapy, serving as a bridge to treatment by offering support and educational resources while they awaited professional help:

So many people are on waiting lists for such a long time...if there was even some nutritional education in an app, that would help.

Therapist 7

Therapists suggested it could assist with helping clients stay engaged between sessions:

They can use the app to follow up their homework...maybe set a goal for the week and the therapist can track their improvement.

Therapist 3

Many viewed an app’s potential as a tool for relapse prevention and self-monitoring after formal therapy has ended:

Once you’ve left treatment...an app could help you check in with your recovery, set goals and face fear foods.

Therapist 4

#### Potential Safety and Efficacy Concerns

Concerns regarding the use of an app for ED treatment fell under safety and efficacy. First, therapists questioned whether an app could ensure a safe and secure environment for users, addressing issues such as data privacy, crisis management, and safeguarding individuals who may be at risk. Second, they were skeptical about an app’s ability to support a meaningful therapeutic relationship, emphasizing the importance of emotional connection, personalized support, and the dynamic, responsive nature of face-to-face therapy for it to be effective.

#### Safety and Security Concerns

Therapists were concerned about how effectively the app could be moderated to prevent triggering or harmful content. In person, professionals could intervene immediately if a conversation became harmful, but an app may lack the same level of real-time oversight. For immediate safeguarding support for someone in crisis, ensuring safety could be difficult without direct therapist support:

One issue came up around like safeguarding, you know again it’s sometimes a bit more difficult to support people particularly if there is a safeguarding issue. You know, if they’re on an app and as opposed to if they’re like in person coming for support.

Therapist 11

Therapists suggested safety concerns could be mitigated by personalizing the app so that triggering content could be hidden or by having a link to a crisis number. There were also worries about users encountering unmoderated discussions that could normalize disordered behaviors or reinforce negative self-perceptions. For discussion forums, strong moderation was seen as vital to maintain a safe and supportive space.

Strong concerns were expressed about data privacy, particularly around sharing sensitive information or ensuring that third parties handling the data were trustworthy and not using data for commercial gain:

I don’t think I would trust any third party with any of the resources that I give out if it was capturing any of their data.

Therapist 7

Concerns were raised by 2 therapists about individuals becoming overly reliant on their phones, which was not perceived always to be healthy, particularly if they were engaging in social comparisons on social media or looking up dietary advice. For these people, directing them to an app could potentially reinforce negative behaviors related to phone use:

Quite often with my clients, they are working on moving away from their phones.

Therapist 12

#### Perceived Ability of an App to Be Effective

Some therapists expressed doubt over an app’s potential to mirror the therapeutic relationship to meet an individual’s needs or to engage users over time. Some questioned the potential to provide the depth and emotional connection that formed part of the therapeutic relationship, which was central to achieving successful outcomes. Another perceived challenge was how an app could deliver a treatment experience that aligned with person-centered therapy and be adaptive to the unique needs of individuals. Some provided suggestions, although these seemed fairly limited compared to what could be achieved via face-to-face therapy:

Having some question prompts at the beginning to try and uncover what that person will relate to more could be helpful.

Therapist 12

Therapists also noted that maintaining user engagement over time may be difficult due to factors such as the lack of sustained motivation and the absence of real-time human interaction. However, they suggested that reviewing progress, gamification, personalized feedback, and celebrating small wins would be beneficial within an app:

If you want to look back at what you’ve recorded in week one compared to where you are now, how are things different and even just ask them what have you learned, you know, over the last week or two?

Therapist 11

You could have different story modes. So people finish one, they start anew. And so that will keep engagement.

Therapist 9

### Theme 5: Suggestions for App Content and Design

#### App Design

Therapists recommended that an app should provide clear guidance on its purpose and benefits to users, along with personalized content ideally adapted to suit individuals’ learning preferences. Design recommendations focused on ensuring the app was user-friendly and engaging, including a simple, intuitive interface with easy navigation and avoiding too much text. Reminders and notifications could be helpful to prompt users to engage with activities, such as food and thought diaries, ensuring these were timely and relevant but not overly intrusive. Rewards and gamification were suggested, with options for unlocking new content or incentives, explicitly avoiding rewards related to changes in eating behaviors. Safeguarding support was mandatory with immediate access to crisis support and coping skills.

Personalization options, such as allowing users to add their names, choose themes, and customize their home pages, were recommended to enhance user engagement. In addition, incorporating fun and light-hearted elements was suggested to make the app more inviting and enjoyable, helping to sustain user interest and participation similar to traditional therapy.

#### App Content

The therapists used various tools, such as CBT worksheets, food journals, and mindful eating exercises, to engage and support clients. These tools were integral to the therapeutic process, offering clients practical strategies and support as they worked toward recovery. Therapists filtered out those they thought were suitable for app inclusion and those that would be more appropriate for therapist-led activities. Although there was a good level of agreement, some therapists were more cautious about the ability of an app to support certain activities, such as exposure therapy. Table 1 presents a summary of key tools used and their perceived suitability for an app.

**Table 1:** Therapists’ perceptions on which therapeutic tools might be transferable to an eating disorder app.

Therapeutic content and example tools		Appropriate for an app	Unsure of whether transferable
**About CBT^a^**			
	Five areas model (“hot cross bun model”)	✓	
**Psychoeducational content**			
	Set-point theory	✓	
	Food-related myths	✓	
	Consequences (health, social, and emotional)	✓	
**Understanding your emotions**			
	Emotion wheel and mood cards	✓	
**Coping strategies and tools**			
	Safe space visualization	✓	
	Breathing exercises (eg, box breathing)	✓	
	Grounding exercises	✓	
**Self-monitoring tools**			
	Food and mood diary	✓	
**Thought records and cognitive restructuring**			
	Thought records	✓	
	Challenging negative thoughts		✓
**Goal setting and tracking**			
	SMART^b^ goal framework	✓	
	Action plans	✓	
**Exposure therapy**			
	Fear ladder		✓
	Behavior experiments		✓
**Distraction techniques**			
	Urge surfing	✓	
	Distress hills	✓	
	Trigger toolbox	✓	
**Motivational tools**			
	Motivational quotes	✓	
	Praise and positive affirmations	✓	
**Self-esteem work**			
	Value-based work		✓
	Strengths and qualities		✓
**Body image work**			
	Body acceptance activities	✓	
	Behavior experiments		✓
**Social support**			
	Peer stories and recovery stories	✓	
	Online community		✓
**Gratitude journal**			
	Gratitude journal	✓	

^a^CBT: cognitive behavioral therapy.

^b^SMART: specific, measurable, achievable, relevant, time-bound.

Psychoeducational support was deemed essential and valuable, offering information on nutrition, the health impacts of ED behaviors, and debunking common food-related myths. Coping strategies, such as breathing exercises, safe space visualization, and grounding techniques, were also recommended. The app could usefully introduce the fundamentals of CBT and how it worked and incorporated practical tools, such as food and mood diaries, goal setting, and recording negative or automatic thoughts. Exposure techniques, such as fear ladders were mentioned, though there were concerns about suitability as a self-guided activity. Distraction methods, including urge surfing and distress management tools, were considered important and highly suitable for app inclusion. Therapists also emphasized the need to address body image and self-esteem, suggesting that selected content be integrated throughout the app, though therapist support may be required for more challenging work.

## Discussion

### Principal Findings

This study advanced our understanding of how to design and develop clinically safe, evidence-based ED apps that can complement therapy by extending continuity of care and the self-management and psychoeducation of clients.

Therapists emphasized an integrative, adaptive CBT that incorporated elements of other therapeutic approaches based on individuals’ needs and the requirement for strategies to support engagement and motivation within an app [27]. Digital ED interventions based on CBT have previously been developed in combination with other theoretical approaches, such as ACT [28], suggesting an integrated approach to treatment can be effective [10] and could be further extended.

Therapists’ resistance to incorporating digital interventions into their treatment could be softened with content that mirrors actual practice and mitigates key concerns, such as the lack of trust, low credibility, and the lack of evidence base perceived in existing apps. Support, but not replacement, of the existing therapeutic relationship was one of the major issues. Some elements of a supportive design could be achieved through personalization, interactive features, and a language style that aims to replicate elements of the therapeutic relationship, such as trust, empathy, and developing bonds [29]. The term “digital therapeutic alliance” defines the alliance a user might perceive with a digital intervention, transferring concepts from a traditional face-to-face therapeutic alliance [30], while also considering the strengths afforded by this medium. For example, just-in-time interventions, which predict and intervene at the point of need, may be one way in which a digital therapeutic alliance could be facilitated [21]. Another way includes building empathy with the user, such as sharing relevant case studies, peer support [31], and the use of affirmative, supportive language [32]. Providing regular, person-centered communication through reminders, check-ins, and motivational messages [33] can also make users feel more involved and connected to an intervention [34].

However, the limitations and challenges of translating therapeutic elements into an app context are significant. Unlike therapists, an app is unlikely to build the deep empathy and trust needed for a therapeutic alliance. Some progress has been made through the application of AI tools, which have demonstrated an ability to encourage self-reflection [35] and some potential for recognizing and validating user emotions [32]. However, apps are currently limited in their ability to provide real-time, nuanced emotional support, which is a core component of the therapeutic alliance. Concerns regarding privacy, safety, and security mean any integration of AI tools within apps requires user involvement, ethical discussions, and extensive testing in advance [36].

Customizing the app’s resources, such as providing a range of multimedia formats to accommodate different user needs (eg, ADHD or autism) and catering for the specific needs of male individuals and ethnic groups, is key to maintaining engagement [37] and inclusivity. The use of reminders and prompts could support engagement so long as they are relevant, supportive, and could be turned off, consistent with a previous study on users’ app preferences [7]. Offering tailored visual elements or customizable content, such as a personal dashboard, can make users feel more connected to an app, similar to how therapists personalize their approaches to individual clients.

The collaborative aspect of therapy, where clients work with therapists to set goals and navigate challenges, suggests that an app should emphasize user agency [38]. It could achieve this by providing users with the ability to set personalized goals and offer feedback on their progress, encouraging users to take ownership of their recovery [39]. The app might include features, such as progress tracking, feedback loops, and reflective exercises, to help users remain aware of their journey, fostering motivation through visible achievements no matter how small. These small achievements should be celebrated on the way, providing further motivation for the user. Importantly, setbacks should also be managed within the app so that these are not seen as a failure but as a learning opportunity, as in therapy. Rewards could be used, though only in connection with activities that help them progress, such as completing journal entries, and not in relation to food or changes in eating behavior, which could be counterproductive. Working closely with therapists during development helps ensure the app is designed in alignment with therapeutic goals and can provide digital support that complements face-to-face treatment.

Although therapists provided suggestions for app content and design based on their therapeutic practice, apps have the potential to extend beyond therapists’ expectations. For example, an app could enhance therapy and improve user understanding of key concepts, such as the five areas model [40] (“hot cross bun model”), through an interactive, step-by-step format, making the content more engaging and accessible for users. Apps could also support more challenging activities such as exposure therapy by providing a structured, supportive, and step-by-step approach to help users confront and overcome their fears. This process could be integrated with therapist support, allowing clinicians to monitor progress, provide guidance, and adjust exposure tasks through the app interface. There is the potential for a paradigm shift in how therapists perceive apps and their potential value if they are developed using evidence-based methods and in consultation with therapists and users.

It was agreed that an app could complement therapy across various stages of the treatment pathway, including before the beginning of the therapy, during the therapy as a supplementary tool, and after the therapy to support recovery, broadening their potential scope. A recent meta-analysis supported this “blended approach,” with patients who used a mobile app alongside therapy having better outcomes than those who did not [41]. Assessing readiness for [42] change before or early within the intervention may be important, as it determines whether a person is prepared to engage or requires an alternative form of support. It is imperative that the safety and security concerns of therapists are addressed within app development.

The key recommendations are mentioned subsequently.

The first recommendation is to comply with data privacy regulations (eg, General Data Protection Regulation and Health Insurance Portability and Accountability Act). Transparent communication about how user data will be used, stored, and protected is fundamental to building trust during early interactions with the app, providing an environment that is safe and secure for users.

The second recommendation is to communicate who the app is for. Apps should clearly specify that they are aimed at individuals with mild to moderate EDs, those in early stages, early recovery, or for relapse prevention, which could include step-down support for more severe patients who are in recovery.

The third recommendation is the immediate access to safeguarding support, that is, providing immediate access to crisis support either to a therapist or to an emergency service. Provide a “panic button” for immediate access to personalized coping skills.

The fourth recommendation is to manage triggering content. Users will need to be able to customize their content, block triggers, and receive warnings before sensitive material content is shown, allowing them to choose whether to proceed or skip. Therefore, understanding potential triggers in advance is crucial to ensure sensitivity around content, requiring thorough testing with users and clinicians before implementation.

The fifth recommendation is a hybrid approach where the app should complement therapy, providing summary reports or personalized feedback to therapists to enhance the therapeutic process. Therapists can address immediate concerns and integrate the app as appropriate to maximize their efficiency and provide between-session support. Therapists should handle complex tasks requiring a therapeutic relationship, such as deep emotional work or anxiety-inducing activities, while managing the overall treatment plan.

The sixth recommendation is app co-design. Collaborative co-design with therapists and users is essential to ensure a person-centered approach to app development, resulting in an app that is clinically suitable, usable, and has the potential to be effective.

### Fit With Existing Research

The concerns about safety and security echo the findings of previous research on barriers to using digital interventions within routine clinical care [13], highlighting the need for data security and crisis support within apps as well as the need for evidence-based psychoeducational content [43]. Skepticism about how well digital interventions could replicate the therapeutic relationship or be able to personalize the users’ experience were also consistent with previous research [23]. However, this study builds on and complements previous research by providing a deeper exploration of the clinical practice of therapists, identifying techniques and strategies that could be implemented in apps to help overcome these challenges. It paves the way for the design and development of more engaging and effective apps that can complement therapy across the treatment pathway.

### Strengths and Limitations

Clinician-centered design enables interventions to be user-centered, clinically relevant, and more likely to be accepted by practitioners. Our study directly engaged with therapists’ concerns, contributing to the development of a safer, more effective, and more engaging app. We have provided detailed and practical recommendations for app content and ensured alignment with current therapeutic practices. This study has highlighted the potential of an app to complement existing treatment for mild to moderate EDs and improve therapists’ efficiency and patient outcomes. This level of specificity increases the likelihood that future digital interventions will include features that benefit both therapists and their clients. Although the study included only 12 participants through a convenience sampling method, we found, despite differences in experience, training, and qualifications, significant overlap during the coding process, which contributed to the creation of consistent and coherent findings.

### Further Work

App co-design work with users and therapists can advance these ideas through a process of iterative development and user testing. Focus groups of potential users ensure the app’s personalization features and motivational tools are usable and have the potential to be effective. Collaborating with therapists will ensure the app aligns with therapeutic best practices, including the co-design of specific features (eg, fear ladders) to make sure they can be safely implemented for self-management and to identify when therapist involvement is required. An exploration of how a blended approach would work in practice is also required in terms of how to integrate an app with therapist sessions.

Integrating and evaluating the suggested components using mixed methods will allow for measurement of their impact on user experience, adherence, and clinical outcomes. This should be completed across a diverse range of participants in terms of sociodemographics and ED experiences. Pilot studies are needed to test app efficacy and safety before real-world implementation, along with postimplementation evaluation to assess effectiveness in practice. This work has the potential to develop usable and effective ED apps that are developed to support the treatment of EDs alongside therapy. It is important that it should capture the perspectives of diverse populations, including those with different ED symptoms, life circumstances, cultural backgrounds, and neurodiverse conditions, to ensure that apps are accessible and relevant across a broad range of users.

The therapeutic alliance was confirmed as central to engagement and a predictor of clinical outcomes and is also a key area for research in digital interventions [29]. More work is needed to understand how apps can better support or possibly replicate the emotional depth, trust, and empathy that are central to therapist-client relationships. Further work is needed around digital technology [7], including developing just-in-time adaptive interventions and using AI tools while addressing challenges around patient safety, data security, and AI or chatbot limitations regarding understanding and responding to human emotions [32]. Emerging technological innovations are capable of delivering in-the-moment interventions tailored to individual needs [15], which could enhance the user experience and improve treatment outcomes of ED apps if privacy and safety are paramount.

### Conclusions

This therapist-centered approach paves the way for safer, more effective, and user-centered apps, which have the potential to play a valuable role in ED treatment alongside therapy.

## Appendix

Multimedia Appendix 1 Interview guide.

Multimedia Appendix 2 Baseline characteristics.

Multimedia Appendix 3 Treatment stages.

## Abbreviations

ACT: acceptance and commitment therapy
ADHD: attention-deficit/hyperactivity disorder
AI: artificial intelligence
CBT: cognitive behavioral therapy
COREQ: Consolidated Criteria for Reporting Qualitative Research
ED: eating disorder
REDCap: Research Electronic Data Capture

## Data Availability

The data supporting this study’s findings consist of qualitative interview transcripts and researcher field notes. Due to the sensitive nature of this information and to protect participant confidentiality, these data are not publicly available. However, deidentified excerpts pertinent to the study’s conclusions can be obtained from the corresponding author upon reasonable request.
