# Supplementary material for: Exploring Therapists’ Approaches to Treating Eating Disorders to Inform User-Centric App Design: Web-Based Interview Study
Source: JMIR Form Res. 2025 May 6;9:e68846. doi: 10.2196/68846 (PMC12093069; doi:10.2196/68846)
Supplement: Multimedia Appendix 1 [file formative_v9i1e68846_app1.docx]

**Multimedia Appendix 1:** Interview guide

**Introduction**

We are looking to design an app to support people with a mild to moderate eating disorders (who may or may not have received a diagnosis) whether they are in the early stages of seeking treatment, would like help to manage their recovery, or may be finding that food is becoming problematic for them.

I am keen to have an open discussion around how you think an app could support the people with subthreshold to threshold eating disorders. I am keen to learn from your experience, there are no ‘right’ answers (assume I have no idea!). I would like to keep this session relatively fluid, based on the information you feel it would be useful to share, whilst ensuring we keep on track with the aim of the session.

I would like to remind you to keep patient confidentiality at all times and that I will interrupt you if I am concerned that potentially identifiable information is being shared.

**Warm-up conversation**

Please could you talk to me a little bit about role as a “*job title*” at “*organisation*” and the types of people who you support and how much experience you have had in this capacity.

Ask them to describe their previous experience of digital eating disorder interventions or apps – what they think they do well, or not so well.

Key topics will include:

| **Topic area** | **Questions** |
| --- | --- |
| **Treatment Approach** | What is your treatment approach when faced with someone with eating disorders?  What theoretical approaches do you apply in treatment? |
| **Process and Structure** | How would you go about applying this approach in practice?  Do you ever deviate from the protocol or introduce elements from other therapeutic approaches?  What is the process you follow in terms structure, including no. of sessions? |
| **Target group** | How do you think an app might be helpful in supporting people mild to moderate eating disorders?  (Prompts: Who do you think would most benefit from an app? |
| **Functionality** | What do you think it will be important to include?  Prompts: resources, psychoeducation etc.  What do you currently provide that you think could be done within the app? |
| **Design** | What are your thoughts on the design?  Prompts: look and feel, layout, navigation etc. |
| **Engagement** | What are your thoughts on how we could make the app engaging for users?  (Prompts: What might it include? How might it be designed/info presented?) |
|  | How could we best engage users? |
| **Motivation** | How do you think could motivate people to use the app? |
|  | How could we encourage them so that they are able to see improvements? |
| **Therapist support** | What resources would you like included, to support you in your role (aimed at the less experience therapists, to make them feel more confident and feel better equipped) |
