# Supplementary material for: Exploring Therapists’ Approaches to Treating Eating Disorders to Inform User-Centric App Design: Web-Based Interview Study
Source: JMIR Form Res. 2025 May 6;9:e68846. doi: 10.2196/68846 (PMC12093069; doi:10.2196/68846)
Supplement: Multimedia Appendix 2 [file formative_v9i1e68846_app2.docx]

**Multimedia Appendix 2:** Baseline Characteristics

**Table 1:** Baseline Characteristics

| Category | Sub-category | Value (out of n=12) |
| --- | --- | --- |
| Age | Mage  Range  SD | 28.7 months  21.4-52.0 years  7.3 years |
| Clinical Orientation | Cognitive Behavioral Therapy  Person-Centred Therapy  Compassion Focused Therapy  Integrative Therapy  Acceptance Commitment Therapy  Dialectical Behavior Therapy  Psychopharmacology | 92% (n=11)  32% (n=4)  32 % (n=4)  16 % (n=2)  17 % (n=2)  8 % (n=1)  8 % (n=1) |
| Role | Psychiatrist  Therapist  Counsellor | 8% (n=1)  58% (n=8)  25% (n=3) |
| Professional Qualifications / Training (please specify) | MA / Msc (related field)  BSc (in related field)  Advanced Diploma | 84% (n=10)  8% (n=1)  8% (n=1) |
| "Digital apps designed to support people with eating disorders may be a valuable tool in the treatment process" | Strongly Agree  Agree  Neutral  Disagree  Strongly Disagree | 42% (n=5)  42% (n=5)  17% (n=2)  -  - |
| "I would likely recommend  a digital app for eating disorders to my clients." | Strongly Agree  Agree  Neutral  Disagree  Strongly Disagree | 25% (n=3)  42% (n=5)  17% (n=2)  8% (n=1)  8% (n=1) |
| 'I have significant concerns about the use of digital apps in the treatment of eating disorders.' | Strongly Agree  Agree  Neutral  Disagree  Strongly Disagree | -  25% (n=3)  42% (n=5)  25% (n=3)  8% (n=1) |
