# Supplementary material for: Exploring Therapists’ Approaches to Treating Eating Disorders to Inform User-Centric App Design: Web-Based Interview Study
Source: JMIR Form Res. 2025 May 6;9:e68846. doi: 10.2196/68846 (PMC12093069; doi:10.2196/68846)
Supplement: Multimedia Appendix 3 [file formative_v9i1e68846_app3.docx]

**Multimedia Appendix 3:** Treatment Stages

| **Stage** | **Description** | **Quotes** |
| --- | --- | --- |
| **Waiting for Treatment** | This section typically involves providing initial support and managing expectations before formal therapy begins. The therapists use this time to prepare clients for the therapeutic journey ahead and address any immediate concerns or anxieties. | *“people who are waiting to receive one to one support and some people then do get one to one support and they carry on attending” (Th9)* |
| **Initial Assessment** | During the initial assessment, therapists gathered comprehensive information about the client's history, symptoms, and goals. This process included both standardized measures and interviews to understand the client's unique situation. A thorough assessment is conducted to gather a comprehensive understanding of the client's history, current symptoms, and therapeutic goals. | *"Initial assessment with them. So these are conducted by a lot of members of staff…and talking about what the main behaviours are, the thoughts and emotions that are behind that as well." (Th8)*  *"We use the long one (EDE-Q) and it’s it goes on about various different factors. Probably takes 20 minutes half an hour most of the time." (Th12)* |
| **Onboarding and Psychoeducation** | Familiarizing clients with the therapeutic process, setting expectations, and beginning to build a rapport. This included core psychoeducation about eating disorders, nutrition and the therapeutic approach (CBT). This content aimed to enhance the client's understanding of their disorder, its impacts, and the rationale behind therapeutic strategies.  Therapists often provided detailed information about nutrition, including the roles of different food groups and nutrients in maintaining physical health. This education helped clients understand the importance of a balanced diet and the physiological impacts of their eating behaviours. It also included discussing the physiological effects of purging, binge eating, and restriction to clients understand the immediate and long-term impacts these behaviours can have on their bodies. | *"use that first session as we get to know session so it’s very relaxed. Very chilled." (Th11)*  *" going through things like the real food pyramid. You know what each food group does for us in the body? What in the diet has for example calcium in it or protein in it?" (Th11)*  "*We do some psychoeducation on why we need food, what food is for and things like that” (Th9)*  *"We’ll talk about maybe nutrition, the importance of each food group in the body... and the impact of almost demonizing foods." (Th11)*  *"For example if someone is purging you know talking about the effects of purging on the body and again from like a physical point of view. Same with binge eating. Same with restriction." (Th11)* |
| **Establishing Healthy Coping Mechanisms** | Early in treatment, therapists focused on helping clients identify and implement healthier coping strategies and techniques to cope with their emotions and triggers replacing harmful behaviours (e.g. binge / purge activities). Techniques may include grounding, breathing exercises or distraction techniques (e.g. creating your own self-care jar/trigger toolbox). | *"What they’re sort of mild active coping techniques are and how we can perhaps help them adopt some healthier coping strategies." (Th11)*  *"I think the replacement is really important. You know like replacing that binge eating or like you know not eating at all behaviour with something healthy." (Th3)* |
| **Goal Setting and Action Planning** | Goal setting involves identifying specific, achievable targets for therapy. This can include dietary goals, emotional regulation targets, or broader life objectives. Clients set specific, achievable goals, and the therapeutic plan is tailored to meet these objectives, focusing on both short-term and long-term outcomes. Providing practical advice on how to achieve goals was sometimes offered, such as help with meal planning, to help clients apply their new knowledge to their everyday life. | *"We talk about coping mechanisms a lot at the beginning as well. And then we as we progress, we focus on... introducing a snack and having three more bites at dinner." (Th9)*  "*And then you can use them that knowledge of what’s worked for them before to try and create a plan that’s going to help them." (Th12)* |
| **Challenging Negative Thoughts and Core Beliefs** | In this phase, therapists work with clients to identify and challenge negative thoughts and beliefs, particularly those related to body image and self-worth. The therapy focuses on identifying and challenging negative thoughts and core beliefs that sustain the eating disorder, working towards healthier cognitive patterns. Core beliefs work involves delving deeper into the underlying beliefs that sustain unhealthy behaviours. Therapists helped clients explore and restructure these beliefs to support healthier thinking patterns. | "*We talk about coping mechanisms a lot at the beginning as well. And then we as we progress, we focus on... challenging anxious thoughts." (Th9)*  *"We challenge. So even if they do gain weight... actually it’s OK for our wait to fluctuate*." *(Th4)*  *"Looking at the deeper kind of self-esteem issues all of those things core beliefs and yeah and just trying to give more sense to those and provide a bit more compassion*." *(Th8)* |
| **Behaviour Experiments** | Therapists encourage clients to test new behaviours in a structured and safe way. This helps clients challenge their fears and develop confidence in managing their behaviours. A couple of therapists described a “step ladder approach” to address fear foods, which is a specific application of a fear ladder in the context of eating disorders. This process of graded exposure can induce significant anxiety, so the therapist provides support and use of coping strategies, such as relaxation techniques or cognitive restructuring, to help manage this anxiety to enable them to progress through the ladder. Clients are encouraged to experiment in a safe and structured manner, gradually challenging their fears and building confidence. | "*Like food challenges specifically with restrictive eating and offered so like step ladder approach... So first of all, I would have a fake away. No, I would make a pizza myself at home because it’s a little bit of a scary food. But I’m in control. That’s OK." (Th4)* |
| **Body Image** | Specific interventions are designed to address body image issues, helping clients develop a more positive and realistic view of their bodies. *Therapists also often* educated their clients on the influence of social media and diet culture on body image and self-perception. This education aims to develop critical thinking skills regarding media messages and societal standards of beauty.  Therapists explain set point theory, which suggests that each person's body has a natural weight range it strives to maintain. This helps clients understand weight fluctuations and counteracts misconceptions about dieting and weight control. | *"We obviously do a lot of work around body image challenging thoughts around their body." (Th11)*  *"It’s like a body image perspective... getting them to just write down areas of their body that they’re not happy with at the moment and actually just asking questions like OK what does that body part do for you?" (Th12)*  *"I also delve quite a bit into like the impact of social media and diet culture on body image because that seems to be quite a big pressure a lot of people face that come to us with support” (Th11)*  *"Another bit of psychoeducation I tend to do is covering set point theory. So looking at the impact that genes have on our body image or body shape and weight." (Th11)* |
| **Relapse Prevention** | Relapse prevention involved preparing clients for potential setbacks by identifying triggers and developing strategies to maintain progress and creating a maintenance plan and continuing to support networks. As therapy progresses, the focus shifts to preparing clients for potential setbacks, developing strategies to maintain progress, and creating a relapse prevention plan.  The importance of adopting a holistic perspective was also emphasized, looking beyond the symptoms of the eating disorder to address broader aspects of the client's life and well-being. This is approach is recommended in supporting long-term recovery and resilience, by making sure clients realise and remember what recovery has given them. | *may put on weight but you’re gonna put on weight until your body feels healthy you know." (Th9)*  *"Particularly in the last session of service users. We do a bit of like a maintenance plan so getting them to think OK what was most useful what’s really helped but also what hasn’t and OK if it hasn’t what can I do to change that going forward?" (Th11)*  *"We tend to do we look at like a bit of a recovery plan sort of thing or a relapse prevention plan." (Th8)* |
